# Towards bio-inspired artificial muscle: a mechanism based on electro-osmotic flow simulated using dissipative particle dynamics

**DOI:** 10.1038/s41598-021-81608-7

**Published:** 2021-01-26

**Authors:** Ramin Zakeri

**Affiliations:** Department of Mechanical Engineering, Shahrood University of Technology, Shahrood, Iran

**Keywords:** Engineering, Biomedical engineering

## Abstract

One of the unresolved issues in physiology is how exactly myosin moves in a filament as the smallest responsible organ for contracting of a natural muscle. In this research, inspired by nature, a model is presented consisting of DPD (dissipative particle dynamics) particles driven by electro-osmotic flow (EOF) in micro channel that a thin movable impermeable polymer membrane has been attached across channel width, thus momentum of fluid can directly transfer to myosin stem. At the first, by validation of electro-osmotic flow in micro channel in different conditions with accuracy of less than 10 percentage error compared to analytical results, the DPD results have been developed to displacement of an impermeable polymer membrane in EOF. It has been shown that by the presence of electric field of 250 V/m and Zeta potential − 25 mV and the dimensionless ratio of the channel width to the thickness of the electric double layer or kH = 8, about 15% displacement in 8 s time will be obtained compared to channel width. The influential parameters on the displacement of the polymer membrane from DPD particles in EOF such as changes in electric field, ion concentration, zeta potential effect, polymer material and the amount of membrane elasticity have been investigated which in each cases, the radius of gyration and auto correlation velocity of different polymer membrane cases have been compared together. This simulation method in addition of probably helping understand natural myosin displacement mechanism, can be extended to design the contraction of an artificial muscle tissue close to nature.

## Introduction

### The state of the art

Undoubtedly, one of essential issues in nature is the movement of living organisms in the sea, land and air. Muscle contractions are responsible for causing this displacement^1^. How muscles contract in the deepest layer, are almost entirely related to the contraction of micro filaments and within microfilaments are associated to the two vital organs myosin and actin. The most important part of the contraction depends on the movement of myosins, which they act like the movement of paddles and causes wavy movements and contraction in a microfilaments is formed. Combinations of these movements provide fiber and muscle contraction^1–3^. The movement of myosin probably originates from the release of calcium ions, fluid transfer, and the release of energy, which is caused the swelling of thin membrane and motion of myosin should be formed while exactly mechanism of myosin motion is one of the unknowns in physiology. Based on the valid sliding theory of muscle contraction, with angular movement of a myosin, the filament contraction will be occurred and consequently contraction of fibers and muscles will be resulted considering the combination of series and parallel mechanism of filaments^1,4,5^.

In order to imitate nature and fabrication of artificial muscles for providing a displacement^6^, various works have been reported including ionic polymer metal composite^7,8^, dielectric elastomer^9^, ionic conducting polymer^10^. These methods need high voltage to operate or the efficiency or mechanisms of these methods are not like a natural muscle contraction^1^. Also, the independent methods to high voltage such as shape memory alloy^11^, molecular machines motion^12^ or polyvinyl chloride gel^13^ need more time for reaction compared to real natural muscle. Due to the many limitations of the proposed methods compared to natural muscle contraction, there is a requirement to study a natural muscle more closely and the function of a small artificial muscle should be simulated by imitating nature.

Since one of the main elements in myosin displacement is fluid and ion transfer, the proper method to perform this process for manufacturing an artificial muscle like a natural muscle requires a suitable and controllable method to pump nano/micro flow^14^. In various industries or research works for pumping micro-flow, electro-osmotic flow method is one of the most practical and suitable methods^15–19^. Based on the electro-osmotic phenomenon, when an electric field is implemented across the micro or nano-channel, due to the electric double layer in the channel wall, the fluid tends to move and this type of pumping flow is called electro-osmotic flow^20^. Smoluchowski^21^ was the first person to provide an analytical solution for a Newtonian fluid in an electrostatic flow in a simple channel. Patankar and Hu^22^ proposed a numerical solution for complex geometries in electro-osmotic flow. One of the problems in micro-scale flow simulation is the discontinuity of the fluid, which requires its own method for simulation. Using Navier–Stokes equations with assumption of fluid continuity cannot accurately represent all existing fluid phenomena in nano/micro scales^20,23^.

According to molecular simulation methods in nano/meso/micro scales, the classical dynamic molecular method has been used in various cases such as electro-osmotic flow simulation in nano scale^24^. It should be noted that at the meso/micro scale, the molecular dynamic method is not a suitable approach due to the high computational costs^25^. Suitable methods are LBM and DPD methods^25,26^. Boyd et al.^26^ used the LBM method to simulate electro-osmotic flow in the micro-channel. According to the LBM method, the direction of particle motion is limited in several directions and has a lower degree of freedom than the DPD method^20^. In the DPD method, a cluster of particles is considered as a particle and the collisions of particles with each other are studied like the MD method, which is caused providing higher length and time scales and the computational cost is much lower than the MD method^20,25,27,28^. Gao et al.^29^ used the DPD method to model hydrodynamic and thermal fluctuation effects in Microscopic dynamics of the process of nanofibers production with consideration of shear flow and solvent evaporation effects. Zakeri et al.^30–33^ used DPD method to simulate different applications such as electro-osmotic flow in the micro channel, polymer motion in the micro-channel, investigation of Newtonian and non-Newtonian fluids etc. One of the important applications of DPD method is in simulating complex fluids such as polymer motion in fluid particles or simulating multiple fluids. Cao et al.^34^ applied the DPD method to simulate the polymer-grafted cylindrical nanopore in EOF. They studied the polymer behavior through DPD particles in EOF. Darbandi et al.^33^ showed that the use of electro-osmotic flow in the transmission of monomer is being able to reduce the dispersion of the polymer chain compared to differential pressure methods.

Although there are recently several methods in field of artificial muscle such as sheath run artificial muscle^35^ which is driven electro-thermally or by vapor absorption or a sheath-run electrochemical muscle can provide 40 times stronger than human muscle or 9.0 times the highest power alternative electrochemical muscle. Also, Wu et al.^36^ applied unique structure of pristine phosphorene to demonstrate that this material can provide remarkable displacement, around maximum actuation strain as high as 36.6% that is more than graphene and comparable with natural muscle but these mechanisms far from real nature. In this paper, proposed mechanism is more close to real nature. It seems that by using a polymer membrane and electro-osmotic flow, a mechanism can be achieved that it may work close to the natural myosin mechanism.

### Objective

**Figure 1:**
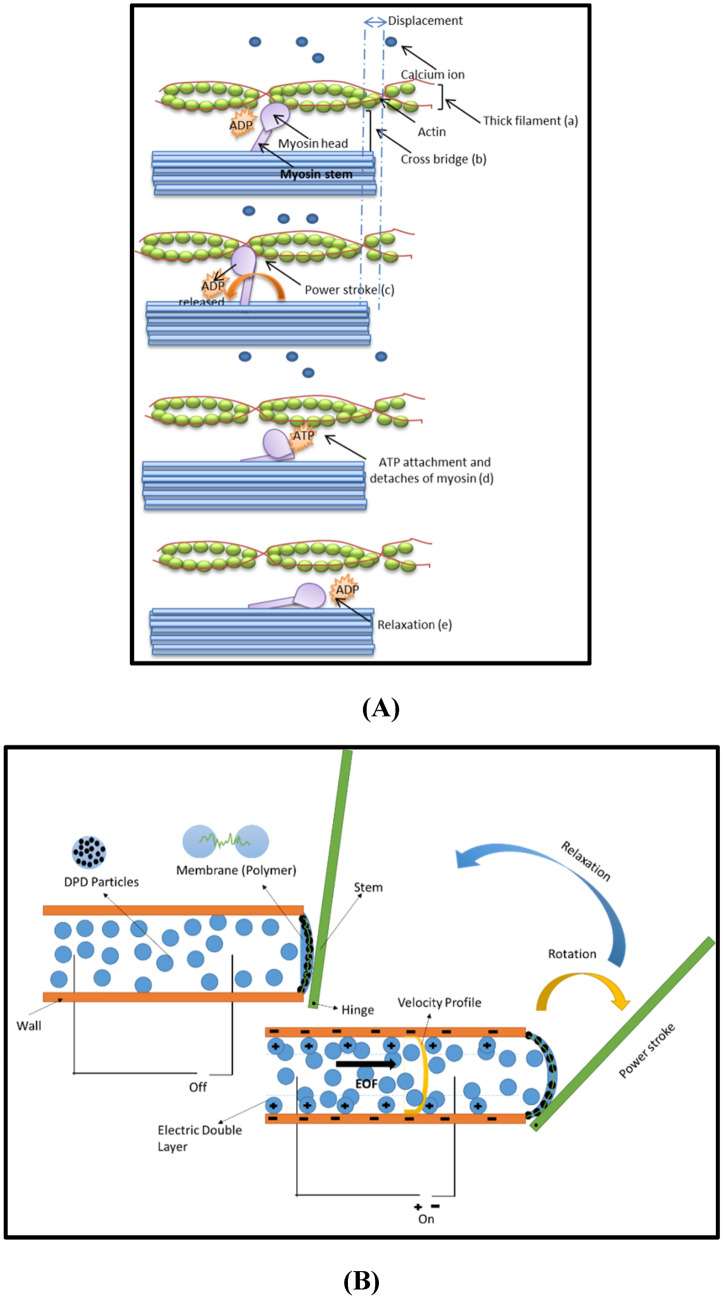
A thin filament of natural muscle mechanism and proposed artificial mechanism. Natural muscle (filament) movement (**A**): action potential (a), bridge connection (b) and power transfer operation (c), separation (d) and relaxation (e)^34^. Artificial muscle movement (**B**): EOF/on and transfer of power to the membrane, stem and angular rotation, also EOF/off and relaxation.

With an overview of the natural mechanism of muscle contraction in nature, almost all living things use a common mechanism for muscle contraction and movement. The contraction mechanism of a real muscle and the present proposal are shown in Fig. 1A. Initially, by stimulating the nervous system, the electrical signal causes the release of calcium ions from sarcoplasmic reticulum and calcium ions cause ATP change to ADP in myosin and the movement of the myosin stem (a). The result of stem movement is the binding to actin, which is called cross bridge (b) and the action of muscle contraction (c). In the opposite direction, with the arrival of an electrical signal to end the contraction process, all the calcium ions are collected in sarcoplasmic reticulum and ATP will connect to the myosin (d), it causes the myosin to move in the opposite direction and the muscle will be relaxed (e)^1–3^.

In the present proposal in Fig. 1B, the electro-osmotic method is used to move the fluid like the transfer of ions in normal muscle (see a). Electro-osmotic is a form of pumping fluid by electric force. The fluid moves under the influence of electro-osmotic flow and causes to stretch the membrane or polymer chain when fluid flow pushes it (see b). The tension of the polymer causes the transfer of power to the hinged stem from one side and the stem will move angularly (see c). When the electric flow cut off, the electro-osmotic force is zero and the fluid moves backwards (see d) and the artificial myosin stem is relaxed (see e). Such a movement is the beginning of a muscle contraction like a real muscle. To simulate this artificial muscle contraction, the three basic topics of electro-osmotic flow, DPD method, and polymer or membrane chain are briefly reviewed in the Sect. 2.

In this paper, according to the natural movement of myosin to contract a muscle, proposed method employs DPD method to simulate EOF and performance of membrane which transmute the fluid pressure to displacement of an artificial myosin as a heart of an artificial muscle. Factors affecting the occurrence of this movement have been investigated in two general categories of factors affecting the electro-osmotic flow and factors affecting the DPD method and membrane.

## Methodology

In order to simulate the mentioned model, it is necessary to check the equations of three sections including electro-osmotic flow, polymer chain, DPD simulation method, which is presented in the following.

### Electro-osmotic flow (EOF)

Normally, without applying electrical field (E0) to a micro-channel, the fluid is electrolytically neutral, but when an electric field is established, the fluid in the channel begins to move as it exits the electrolytic neutral state. This type of movement and pumping of fluid is called electro-osmotic flow (EOF). The root of this motion is in the electric double layer (= 1/k). In other words, because the surface is charged and the electrolyte solution has positive and negative ions and they are affected by the wall, ions of the opposite types should be absorbed by the wall, while ions of the same kind should be disposed of each other. This particular arrangement is often referred to as the electric double layer. Inside this layer and adjacent to the wall, a very thin layer (around a nanometer) is situated which is called stern layer. The ions of this layer are completely attached to the wall and are immobile. As the next layer is mobile layer with a thin thickness around a few nanometers to a few micrometers widths, called the diffuse layer, thus there is a shear layer between them and flow movement will start from this layer. In the diffuse layer, the concentration of ions opposite the wall decreases to be equal to the concentration of ions in the wall^20^.

One of the basic parameters in the arrangement of ions is the electrical potential at the surface or the potential at the interface between the stern layer and the zeta scattering layer is the zeta potential (ψ_0_) and its value depends on the solid and fluid surface of the electrolyte. The fluid on the one hand is affected by the zeta potential and on the other hand fluid is under effect of electric field which consequently will cause the fluid to move. The smaller the thickness of the double layer is resulted from higher ionic concentration of solvent and it will affect on the velocity profile or gives higher the dimensional ratio of kH parameter (channel height $\times$ inversed electric double layer thickness).

The equation that governs this type of motion is Poisson equation which is

1 $${\nabla }^{2}\Psi =-\frac{{\rho }_{e}}{\epsilon }$$

where *ψ* is the electric potential, ${\rho }_{e}$ is net charge density and $\epsilon$ is relative permittivity of the solvent. *ψ* depends on geometry and boundary conditions and net charge density can be extracted from Eq. (1). Considering the electric field of ${E}_{0}$ and substituting in Eq. (2), EOF force can be calculated:

2 $${F}^{e}={E}_{0}{\rho }_{e}$$

This force is applied to all fluid particles and causes the motion and transmission of the momentum. The result of this force is the transfer of force to the membrane and the swelling of membrane, which is explained in the next section on how to simulate DPD particles and membrane simulation^25^.

### Polymer membrane (polymer chain)

The main innovation of this research is in the swelling of membrane and transfer of fluid power to move the artificial myosin. To simulate this thin membrane, it is sufficient to place an impermeable polymer chain in two dimensions across the channel that can hold particles and transmit power. For modeling of a polymer chain, it is sufficient to consider a number of beads and springs attached to them and consequently spring force should be added in conservative force. This force is:

3 $${\mathbf{F}}_{ij}^{p}={S}_{hb}{r}_{ij}$$

where the spring hardness coefficient is $${S}_{hb}$$ or the harmonic bond constant. Two important indicators in the displacement of a polymer chain are considered including the radius of gyration and the velocity auto-correlation function (VACF). The radius of gyration indicator can describe the distribution the dimensions of a polymer chain. The radius of gyration is the root mean square distance ($${R}_{G}^{2})$$ of particle from the center of mass (cm) which is presented as^31–33,37,38^

4 $${R}_{G}^{2}=\frac{1}{n}\sum_{1}^{n}{\left({r}_{i}-{r}_{mean}\right)}^{2}=\frac{1}{{2n}^{2}}\sum_{ij}{r}_{ij}^{2}$$

in which, $${r}_{mean}$$ is the mean position of the polymer chain, $${r}_{ij}=\left|{\overrightarrow{r}}_{i}-{\overrightarrow{r}}_{j}\right|$$ and *n* is the beads number in a monomer. Also, for calculation of VACF, at the first, velocity of the center-of-mass of the polymer chain ($${v}_{i})$$ should be calculated:

5 $${v}_{i}(i{t}_{vacf})={\sum }_{i=1}^{n}\frac{{v}_{i}(i)}{n}$$

where $i{t}_{vacf}$ is iteration number of time step and consequently it will use for updating and label of the register. Then, considering number of time step, the VACF should be updated:

6 $${V}_{vacf}(it)={\sum }_{i=1}^{it}{v}_{i}\left({it}_{t0}\right){v}_{i}(i{t}_{t+t0})$$

where $\left({it}_{t0}\right)$ and $\left(i{t}_{t+t0}\right)$ are the origin time of polymer and summation of origin time with delay time. It should be noticed that polymer chain is not influenced from EOF and motion of polymer chain is resulted from momentum transfer of fluid particles which the fluid motion is caused from EOF^39^.

### Dissipative particle dynamics (DPD)

According to this method, a number of molecules are considered in a cluster and due to the large grain size of the simulation method, the run time of simulation is so faster and the simulation would be carried out in a larger scale than the molecular simulation method and in a smaller scale than the continuous CFD method. The formulation of this method is based on Newton's method in which states the temporal evolution of each DPD particle as a set of molecules/atoms is associated to the velocity of the DPD particles (${\overrightarrow{v}}_{i})$ and consequently the force (${\overrightarrow{F}}_{i})$ applied to each of the particles can be calculated:

7 $${\overrightarrow{v}}_{i}=\frac{d{\overrightarrow{r}}_{i}}{dt}$$

8 $${m}_{i}\frac{d{\overrightarrow{v}}_{i}}{dt}={\overrightarrow{F}}_{i}$$

where $${\overrightarrow{r}}_{i}$$ is position of particle with mass of $${m}_{i})$$. Generally, total inter-particle force ($${\overrightarrow{F}}_{i})$$ consists of two main components including external or EOF force ($${\overrightarrow{F}}_{EOF})$$ in this research and internal forces including **F**_*ij*_^*C*^ or the conservative force, **F**_*ij*_^*D*^ or the dissipative force and **F**_*ij*_^*R*^ or the random force.

9 $${\overrightarrow{F}}_{i}={\overrightarrow{F}}_{external}+{\overrightarrow{F}}_{internal}={\overrightarrow{F}}_{EOF}+\sum_{j\ne i}[{\mathbf{F}}_{ij}^{C}+{\mathbf{F}}_{ij}^{D}+{\mathbf{F}}_{ij}^{R}]$$

10 $${\mathbf{F}}_{{ij}}^{C} = \left\{ {\begin{array}{*{20}l} {a_{{ij}} \left( {1 - \frac{{r_{{ij}} }}{{r_{c} }}} \right){\hat{\mathbf{r}}}_{{ij}} ,} \hfill & {r_{{ij}} < r_{c} } \hfill \\ {0,} \hfill & {~r_{{ij}} \ge r_{c} } \hfill \\ \end{array} } \right. + {\mathbf{F}}_{{ij}}^{p}$$

11 $${\mathbf{F}}_{ij}^{D}=-\gamma {\omega }^{D}({r}_{ij})({\widehat{\mathbf{r}}}_{ij}\cdot {\mathbf{v}}_{ij})({\widehat{\mathbf{r}}}_{ij})$$

12 $${\mathbf{F}}_{ij}^{R}=\sigma {\omega }^{R}({r}_{ij}){\theta }_{ij}{\widehat{\mathbf{r}}}_{ij}$$

where *a*_*ij*_ is the repulsive parameter between particle i and j which can be extended to interaction between fluids, walls and polymers particles, *r*_*c*_ is the cut-off radius normalized to unity, **r**_*ij*_ = (**r**_*i*_*—r*_*j*_), *r*_*ij*_ =|**r**_*ij*_|, and ${\widehat{\mathbf{r}}}_{ij}$ = **r**_*ij*_ /*r*_*ij*_ . Also *γ* and *σ* are the constant coefficients, **v**_*ij*_ = (**v**_*i*_*—v*_*j*_), *ω*^*D*^ and *ω*^*R*^ are weight functions:

13 $$\omega ^{D} \left( {r_{{ij}} } \right) = \left[ {\omega ^{R} \left( {r_{{ij}} } \right)} \right]^{2} = \left\{ {\begin{array}{*{20}l} {\sqrt {1 - \frac{{r_{{ij}} }}{{r_{c} }}} ,} \hfill & {r_{{ij}} < r_{c} } \hfill \\ {0,} \hfill & {r_{{ij}} \ge r} \hfill \\ \end{array} } \right.~$$

*θ*_*ij*_ in the random force is a random function due to meso-scale simulation which has zero mean and unit variance properties. Velocity-Verlet (DPD-VV) algorithm is an applied method to solve equations and update the position and velocity of particles. Also, according to Duong-Hong et al.^25,37^, double layer of frozen particles at the wall with consideration of radius of cut off (${r}_{c}$) and the bounce-back condition are used as a boundary condition implementation on walls.

## Results and discussion

In order to evaluate the obtained results, first, the results are evaluated and validated in a simple channel under the influence of electro-osmotic flow, and then the proposed artificial muscle will be simulated in different conditions.

### Validation

As mentioned in the numerical method section, the electro-osmotic force is formed by implementing the electric field in the fluid, considering the effect of electric charge density and electric field (see Eq. 2), the EOF force in the fluid particles is formed in a double electric layer that starts moving from the walls border and finally the velocity profile is shaped. The final velocity profile is a function of parameters such as ionic concentration (i $\propto$ k) parameter, zeta potential effect due to wall material and E0 or applied electric field.

One of the basic parameters in electro-osmotic flow is the ionic concentration of the fluid. Without ionic property, an electric double layer is not formed and having a minimum value of ionic property is necessary for fluid transfer. According to the Eq. 1 ionic concentration has an inverse relationship with the Debye length, in other words, higher the ionic concentration provides the shorter Debye length and the flatter the velocity profile and vice versa. Dimensionless kH parameter describes the ratio of channel width to Debye length. By increasing the kH parameter, amount of flow velocity is increased to a certain extent and saturation is formed, and after a certain limit, the velocity profile just becomes flatter.

**Figure 2:**
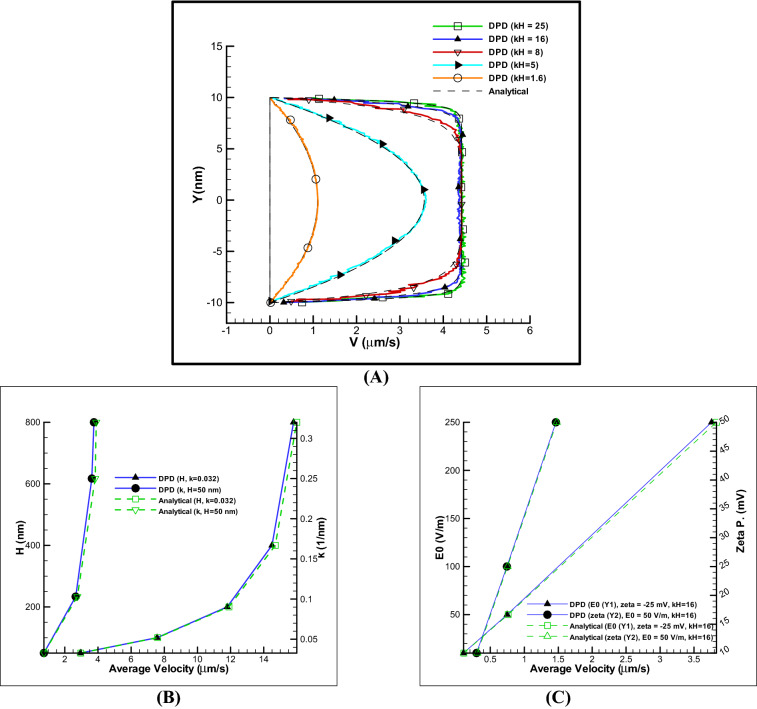
Validation of EOF-DPD results using analytical results. Comparison of DPD method and analytical solution: velocity profiles in different kH parameters (**A**) for E0 = 250 V/m, zeta potential − 25 mV and average velocity influenced from different reverse of EDL (k) and channel height, E0 = 250 V/m, ${\psi }_{0}$=− 25 mV (**B**) and different electric fields and zeta potentials, kH = 16 (**C**).

Figure 2A shows the velocity profile diagram for different values of kH parameters based on the Table 1. As shown, the DPD method is in proper agreement with the analytical results in the simple channel. Also, as the amount of ionic concentration of the fluid increases, the velocity profile becomes flatter and also the velocity value increases to a certain extent, which there is no change in the maximum value but the velocity profile becomes flatter. In the same way in mentioned figure, the average of velocity profiles calculated by DPD method has been compared with analytical results in a wide range of changes in channel height, k parameters (Fig. 2B), electric field, ${\psi }_{0}$ (Fig. 2C). The accuracy of all results is reported with an error of less than 10%. As can be seen, changes in ${\psi }_{0}$ and electric field have a linear relationship with increasing velocity, but the ion concentration non-linearly increases to a certain velocity and no significant change is seen after a certain amount. Also, the validation of polymer chain in nano channel has been reported in ref^31^. It can be concluded that on a particle scale, the DPD method has a proper accuracy in analyzing the complex test cases such as electro-osmotic flow and can be simulated for more complex situations such as simulation of a micro myosin performance in EOF.

**Table 1:** DPD and polymer constant parameters.

DPD-Parameters	Values
*a*_*ij*_^25^	75
*a*_*wij*_^25^	8.66
Channel length	20 $\times$ 20 nm
Particles density	10
Numbers of particles	4000
${\varvec{\sigma}}$	3
${{\varvec{r}}}_{{\varvec{c}}}$	1

Polymer-Parameters	Values
Numbers of beads	50
dt	0.001
*S*	8000

### Displacement of polymer artificial myosin through electro-osmotic nano flow

Fluid transfer is always accompanied by momentum transfer, and if this fluid force is transferred properly, it will be able to move objects. In one classification, changes in polymer motion can be divided into three categories: parameters affected by electro-osmotic flow and parameters affected by fluid and membrane which they are investigated in the following.

#### Impact of EOF parameters

**Figure 3:**
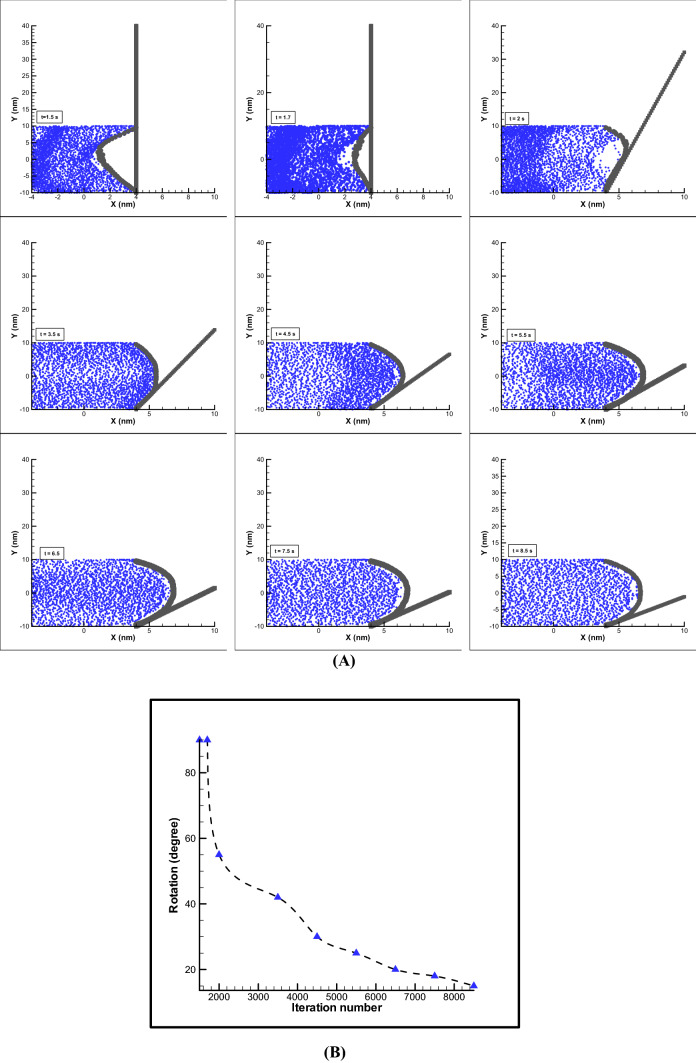
Rotation of stem over time. Displacement of membrane and step due to EOF, kH = 8, E0 = 250 V/m, ${\psi }_{0}$= − 25 mV (**A**). Angular displacement of stem over iteration numbers (**B**).

The electro-osmotic force causes the fluid to move and the fluid enters its force into the membrane and will cause the membrane to swell. Membrane swelling also increases the ability of the stem to move. Placing a polymer membrane at the end of the channel will prevent particles from moving out and the swelling of the membrane will be simulated. Also, by transferring force from the membrane to the stem, it causes the stem to rotate. All the conditions used in the simulation are given in Table 1. The results of this simulation over time are shown in Fig. 3 in various motion forms. As can be seen, with the movement of the fluid by the electro-osmotic force and the momentum transfer of the fluid, swelling is created in the membrane and finally the movement of the polymer and the stem is resulted (Fig. 3A). In practice, this method can be used to create motion with significant displacement, and finally the motion of the solid object displacement can be obtained which angular displacement of stem over iteration numbers is depicted in Fig. 3B.

##### Effect of kH parameter

**Figure 4:**
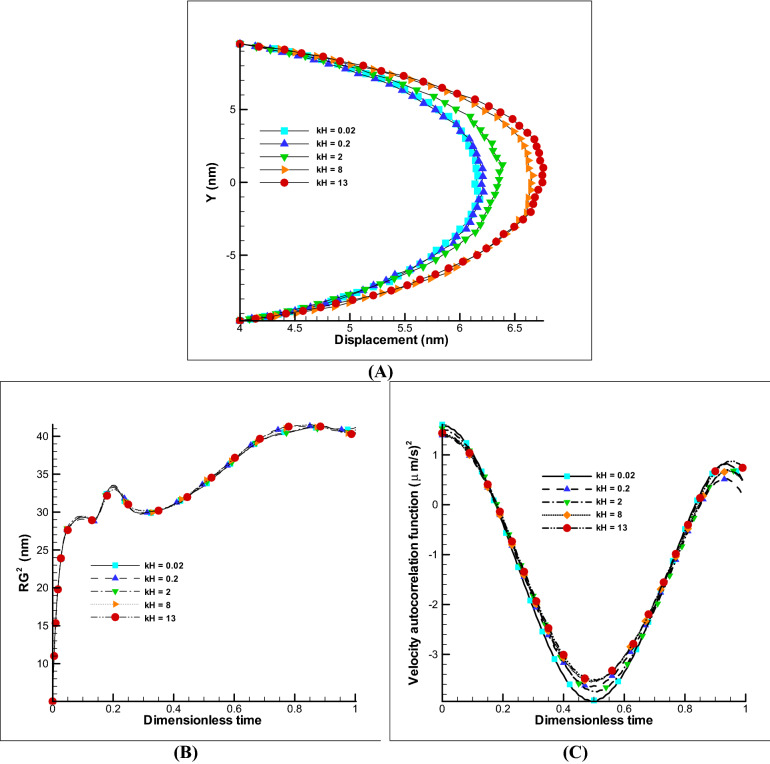
Changes of kH parameter on EOF with E0 = 250 V/m and Ψ_0_ = − 25 mV and impermeable polymer membrane displacement. Different displacement of polymer membrane by changing kH parameter (**A**). Variation of radius of gyration and VACF over dimensionless time (t/t_max_) by changing the kH parameter (**B**,**C**).

Changes in ionic concentration or Debye length (kH parameter) also affects the rate of membrane inflation and will cause different displacements. Figure 4A shows the displacement of the membrane for different kH values. As it is clear, with increasing kH parameter, no significant changes in displacements are observed, and this is a sign of reaching the ionic concentration to saturation condition. Also, the radius of rotation of the polymer or membrane chain has not changed significantly (Fig. 4B) because the displacement rate is not conspicuous. Also, the VACF has not changed so much because there are no significant velocity and dynamics changes (Fig. 4C). It can be concluded that by increasing the ionic concentration of the membrane to a certain extent less than 8% increase in displacement can be achieved and it has a small variation amount in the radius of rotation and VACF.

##### Effect of zeta potential and electric field

**Figure 5:**
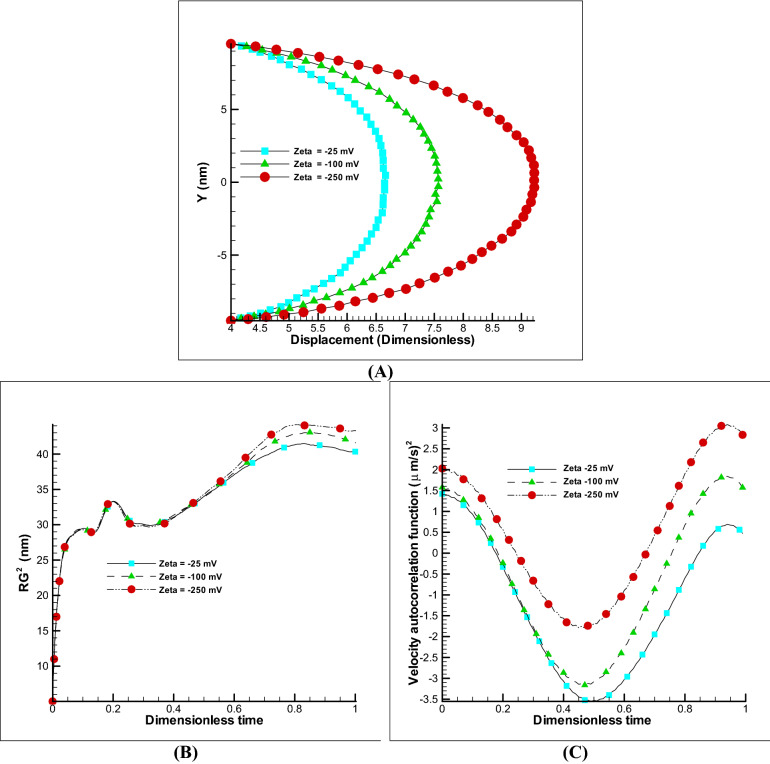
Changes of zeta potential on EOF and polymer membrane. Different displacement of polymer membrane by changing the zeta potential, kH = 8, E0 = 250 V/m (**A**). Variation of radius of gyration and VACF over dimensionless time (t/t_max_) by changing the zeta potential (**B**,**C**).

**Figure 6:**
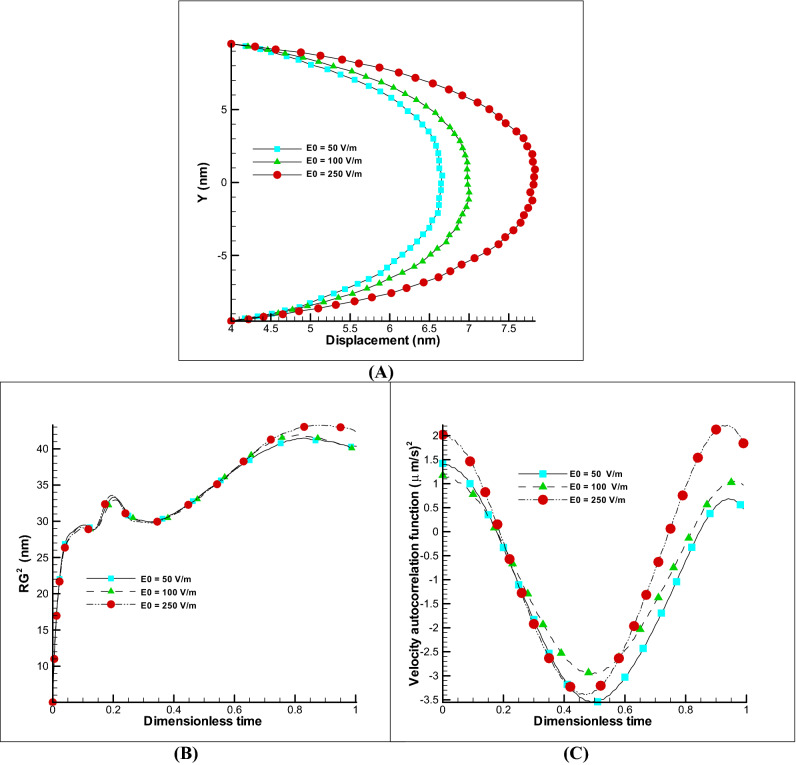
Changes of electric field on EOF and polymer membrane. Different displacement of polymer membrane by changing the electric field, kH = 8, Ψ_0_ = − 25 mV (**A**). Variation of radius of gyration and VACF over dimensionless time (t/t_max_) by changing the electric field (**B**,**C**).

The two electro-osmotic parameters that will have a major impact on the displacement of the velocity profile as well as the polymer chain are the effect of the electric field and the zeta potential. The first parameter can be increased or decreased during movement and is important from the control point of view, while the second parameter is related to the material of wall and affects the strength of the electro-osmotic flow during the construction phase. The changes of these parameters are almost linear on the velocity profile and due to the effect of membrane elasticity; no parameter will have an exact linear effect on polymer membrane. The Fig. 5A shows the effect of zeta potential on the displacement of the membrane. As can be seen, by increasing the zeta potential to ten times, an increasing of about 40% in displacement is obtained, and a significant effect is observed on the radius of gyration and the VACF (Fig. 5B,C). If such a study is carried out on changes in the electric field, we will reach similar results. In Fig. 6A by enhancing the electric field to 5 times, a 23% of increasing in displacement is gained, and a significant effect has been seen on other properties such as the radius of gyration and the VACF (Fig. 6B,C). Note that excessive increase of the field has an electrolysis effect and is one of the limiting parameters in the amount. According to the references^22–25^, the safe ranges were chosen in this paper. It can be concluded that the effect of zeta potential and electric field have a great effect on the displacement of the membrane and in the manufacturing phase the use of high potential zeta materials is appropriate and in the control of membrane displacement, the use of variable electric field is a significant variable.

It is obvious that by studying the ratio of the average velocity of the polymer membrane within 8.5 s and also the average ratio of strain on the elastic membrane in Table 2, it can be concluded that the performance (higher velocity and elongation) for kH more than 8 would be higher than other cases. Also, higher polymer chain performance is related to higher zeta potential and electric field.

**Table 2:** Ratio of average velocity and strain of polymer chain for different kH, zeta potential and electric field parameter.

Parameter $\frac{Vi}{Vj}$ i, j = 1–5 (kH = 0.02. 0.2, 2, 8, 13)	Parameter $\frac{{\epsilon }_{i}}{{\epsilon }_{j}}$ i, j = 1–5 (kH = 0.02. 0.2, 2, 8, 13)
*V5/V1* = *1.28*	${{\epsilon _{5} } \mathord{\left/ {\vphantom {{\epsilon _{5} } {\epsilon _{1} }}} \right. \kern-\nulldelimiterspace} {\epsilon _{1} }} = 1.26$
*V5/V2* = *1.22*	${{\epsilon _{5} } \mathord{\left/ {\vphantom {{\epsilon _{5} } {\epsilon _{2} }}} \right. \kern-\nulldelimiterspace} {\epsilon _{2} }} = 1.23$
*V5/V3* = *1.14*	${{\epsilon _{5} } \mathord{\left/ {\vphantom {{\epsilon _{5} } {\epsilon _{3} }}} \right. \kern-\nulldelimiterspace} {\epsilon _{3} }}$ = 1.17
*V5/V4* = *1.04*	${{\epsilon _{5} } \mathord{\left/ {\vphantom {{\epsilon _{5} } {\epsilon _{4} }}} \right. \kern-\nulldelimiterspace} {\epsilon _{4} }}$ = 1.04

Parameter $\frac{Vi}{Vj}$ i, j = 1–3 (zeta = − 25, − 100, − 250)	Parameter $\frac{{\epsilon }_{i}}{{\epsilon }_{j}}$ i, j = 1–3 (zeta = − 25, − 100, − 250)
*V3/V1* = *1.45*	${{\epsilon _{3} } \mathord{\left/ {\vphantom {{\epsilon _{3} } {\epsilon _{1} }}} \right. \kern-\nulldelimiterspace} {\epsilon _{1} }}$=1.33
*V3/V2* = *1.28*	${{\epsilon _{3} } \mathord{\left/ {\vphantom {{\epsilon _{3} } {\epsilon _{2} }}} \right. \kern-\nulldelimiterspace} {\epsilon _{2} }}$ = 1.23

Parameter $\frac{Vi}{Vj}$ i, j = 1–3 (${E}_{0}$ = 50, 100, 250)	Parameter $\frac{{\epsilon }_{i}}{{\epsilon }_{j}}$ i, j = 1–3 (${E}_{0}$ = 50, 100, 250)
*V3/V1* = *1.95*	${{\epsilon _{5} } \mathord{\left/ {\vphantom {{\epsilon _{5} } {\epsilon _{1} }}} \right. \kern-\nulldelimiterspace} {\epsilon _{1} }}$=1.65
*V3/V2* = *1.45*	${{\epsilon _{5} } \mathord{\left/ {\vphantom {{\epsilon _{5} } {\epsilon _{2} }}} \right. \kern-\nulldelimiterspace} {\epsilon _{2} }}$ = 1.37

#### Impact of DPD fluid and polymer parameters

As it is obvious (see Eqs. 3–12), in addition to the parameters that are affected by the electro-osmotic flow, there are other parameters which they depend on type of fluid, the collision of the fluid with the polymer chain or polymer to polymer. These parameters in this research are divided into four general categories: repulsive parameter or parameter of collision of fluid with polymer and collision of polymer particles with each other, polymer chain length parameter, number of beads used in membrane simulation, particles density or determination of number of particles and stiffness parameter between beads.

**Figure 7:**
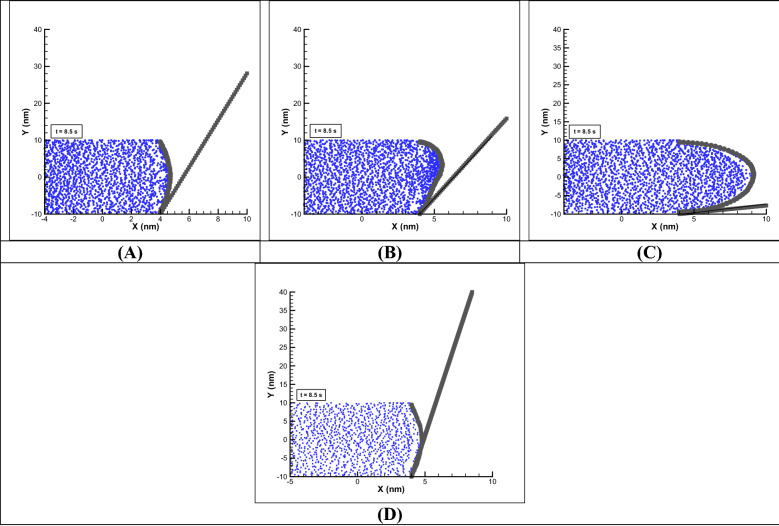
Effect of DPD-polymer parameters on angular rotation of stem compared to Fig. 3 (last frame). Variations of beads number (left to right respectively) from 50 to 30 (**A**), repulsive parameters 4 times (**B**), harmony hardness coefficient 0.5 times (**C**) and particle density 0.5 times (**D**).

In Fig. 7, the effect of the parameters can be understood by comparing Fig. 3, considering kH = 8, E0 = 250 V/m and Ψ_0_ = − 25. Respectively, from left to right by changing the chain length parameter from 50 to 30 (Fig. 7A), almost 30% reduction in displacement, by changing the repulsive parameter between polymer-DPD particles and polymer–polymer particles (see Eq. 10) 4 times (a = 4) around 22% drop in displacement (Fig. 7B), by changing the coefficient of spring stiffness (harmony hardness coefficient) between the beads 0.5 times, about 36% enhancement in displacement (Fig. 7C) and by decreasing the particle density from 10 to 5, about 19% reduction in displacement are observed (Fig. 7D). Obviously, by reducing the number of beads, the intermediate springs between beads are stretched more and the membrane stiffness will be higher and there is less displacement, or by decreasing the stiffness coefficient of the springs, an increase in the length of the springs (strain) will be seen. Also, the changing of the membrane material and type of DPD fluid, using the manipulation of the repulsive factor, the displacement of the membrane is affected and by increasing the repulsion coefficient, less displacement is resulted which these parameters are reviewed and analyzed further in next sections.

##### Impact of polymer beads number

**Figure 8:**
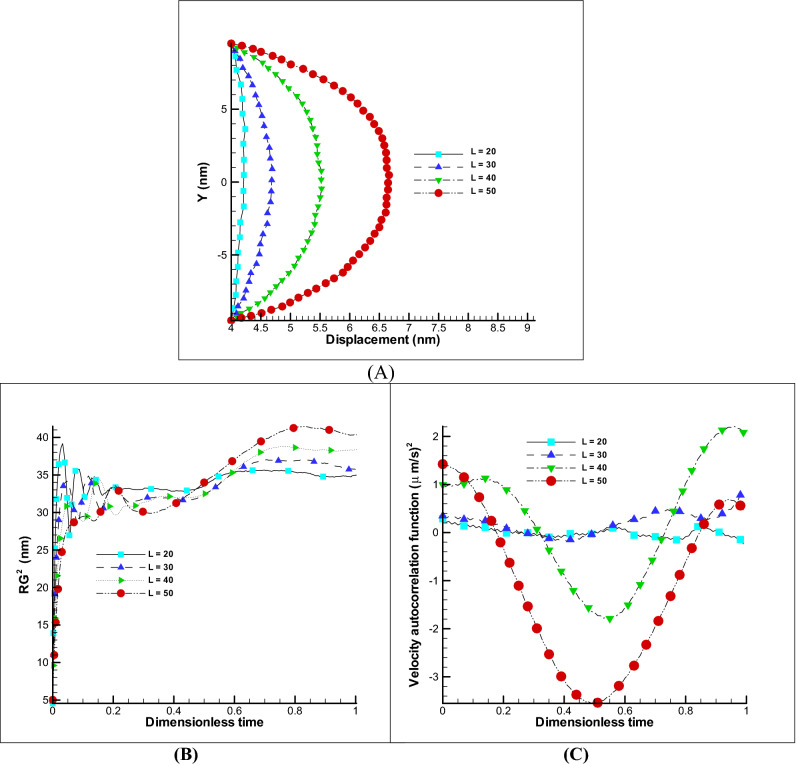
Effect of number of beads on membrane. Displacement of polymer chain in EOF for different beads number (**A**). Changes of radius of gyration and VACF over dimensionless time (t/t_max_) considering different beads number (**B**,**C**).

Since a number of beads and springs are used in the simulation of a polymer membrane, a change in their number will affect the elasticity of the membrane. By reducing the number of beads and considering the beginning and end of the beads are determined and constant, it is clear that the membrane for installation across the channel in the transverse direction should be stretched more and its displacement in the longitudinal direction is reduced. In Fig. 8A change in membrane displacement is clearly observed, and also with a decrease in displacement, a decrease in the gyration radius, and a velocity correlation function (VACF) has been resulted due to less movement (Fig. 8B,C). According to the VACF, it can be detected that a strong nonlinear relationship prevails in the number of fewer beads than the number of more beads. Such a result is also observed in the displacement of the polymer chain in such a way that by increasing the beads from 20 to 30, the displacement changes by about 9% (highly nonlinear trend) and in increasing the beads from 30 to 40 or from 40 to 50, the displacement changes proportional and more which these variations are reported about 20% (low nonlinear behavior).

##### Impact of DPD-repulsive parameter

**Figure 9:**
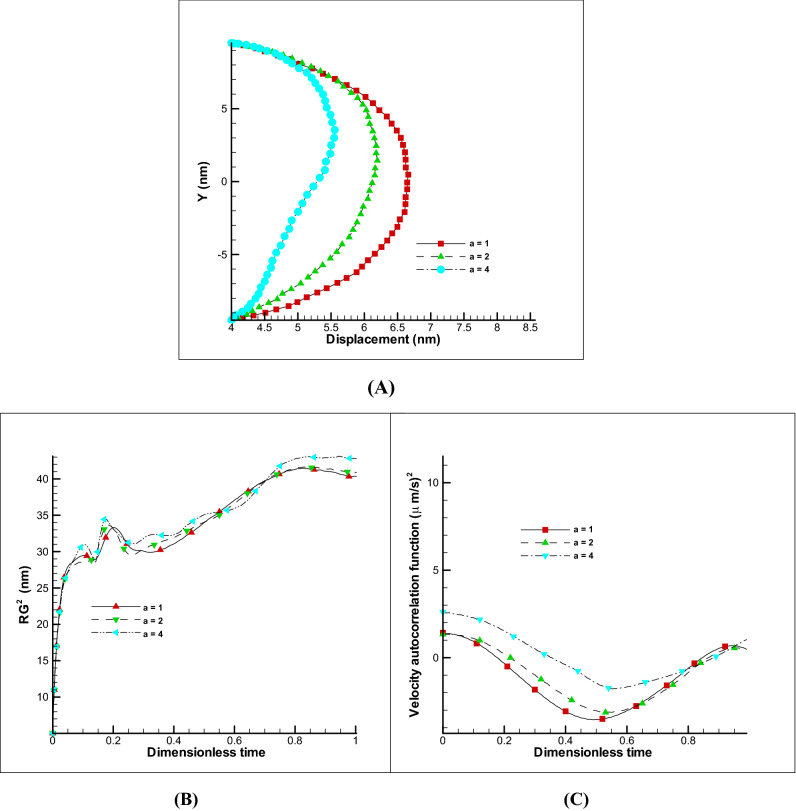
Effect of repulsive parameter on membrane. Displacement of polymer chain in EOF for different repulsive parameter (**A**). Changes of radius of gyration and VACF over dimensionless time (t/t_max_) considering different repulsive parameter (**B**).

The most important parameter for determining the material of the fluid or polymer is the repulsive factor, which is derived from the coefficients of the Leonard Jones equation. In this research, the collision of fluid with polymer and also the collision of polymer with polymer have been investigated using multiplying the repulsive parameter of Table 1 by constant parameter (a). The change of this parameter on polymer displacement is given in the Fig. 9A. As it is known, by changing this parameter (a = 2, 4 (fluid-polymer and polymer–polymer collision)) the maximum variation has decreased by about 16%. As expected, changes in radius of gyration and VACF change slightly (Fig. 9B,C). It can be concluded that depending on the type of fluid used, by changing this parameter, a suitable material of the polymer can be obtained, but the elastic property of the polymer must also be considered, which is examined in the next section.

##### Impact of harmonic bonding parameter

**Figure 10:**
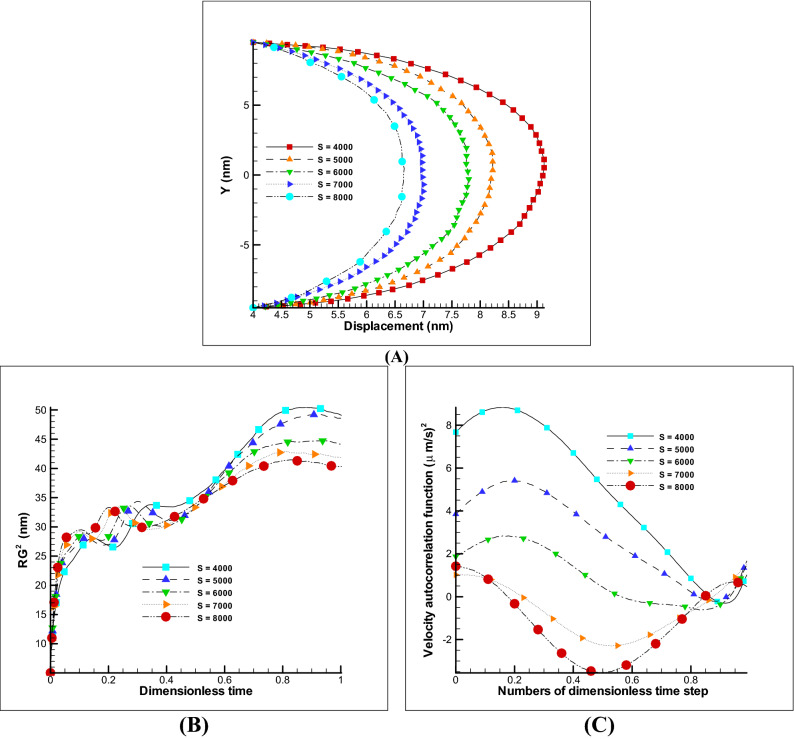
Effect of harmony bond coefficient on membrane. Displacement of polymer chain in EOF for different harmony bond coefficient (**A**). Changes of radius of gyration and VACF over dimensionless time (t/t_max_) considering different harmony bond coefficient (**B**).

The coefficient of elasticity between the beads determines the elasticity of the membrane. Whatever mentioned coefficient is increased, the harder the elongation of the spring and the less displacement will be eventually formed. In the Fig. 10A, for different values of this bond coefficient, which is called by different titles such as harmonic bond coefficient, spring coefficient or elasticity coefficient, is presented. As can be seen, by increasing this coefficient, less displacement is achieved because the resistance force of the membrane against fluid movement would be higher. Considering the changing this parameter as shown in Fig. 10B,C, conspicuous dynamics motion is reported by in investigation of radius of gyration and VACF. In practice, this coefficient has a certain limit and by passing this limit, a notch is formed in the membrane. It can be concluded that this parameter is the most important parameter in determining the amount of elasticity of a membrane and by decreasing the elasticity coefficient to 0.5 times, 36% increase in displacement would be obtained.

##### Impact of density parameter

**Figure 11:**
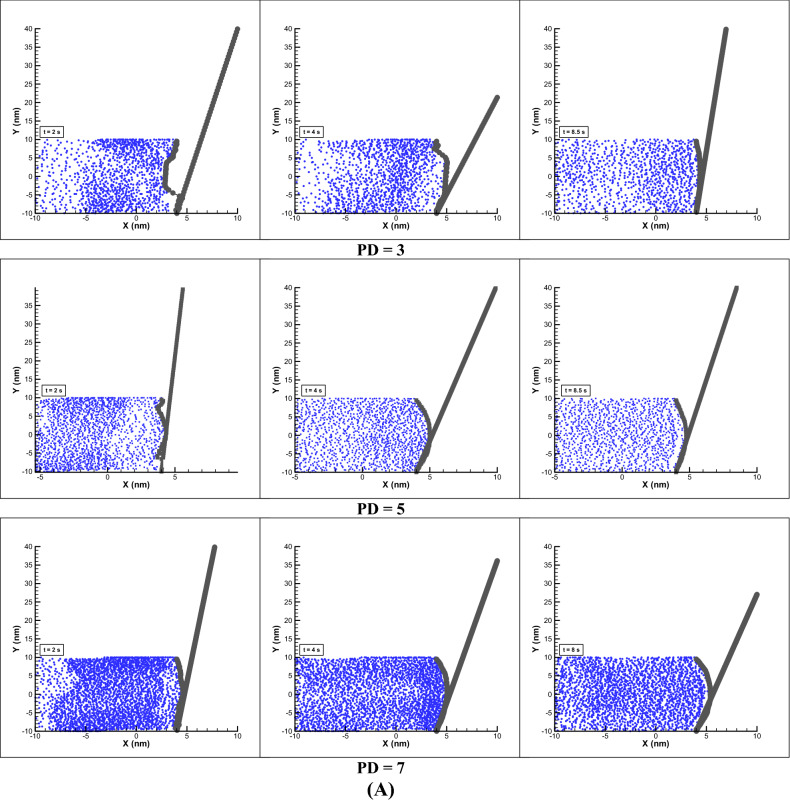

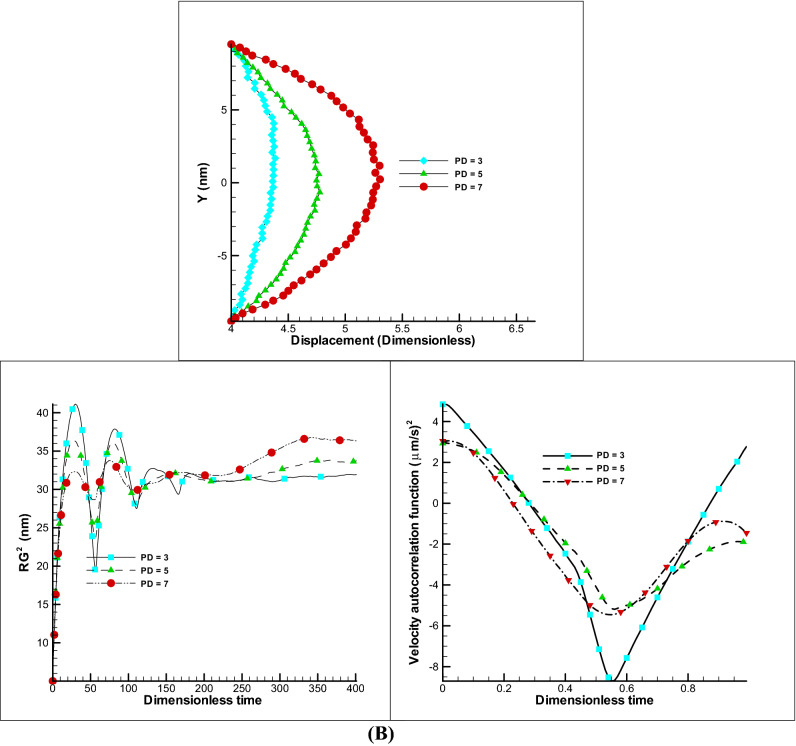
Effect of particles density (PD) on membrane. Displacement of polymer chain in EOF for different PD (**A**). Changes of radius of gyration and VACF over dimensionless time (t/t_max_) considering different PD (**B**).

Since we are dealing with particles and the fluid is not continuum, the particles density parameter (PD) which is related to the number of particles (NP), NP = PD $\times$ DL $\times$ DY in a simulation box (DL $\times$ DY) is very effective in the efficiency of stem movement. In this study, unlike the previous cases where this parameter was 10, in these cases, considering the Fig. 3 conditions, this parameter has been reduced to 3, 5 and 7, and the results of these changes are shown in Fig. 11. Initially, in Fig. 11A, as can be seen, with decreasing the number of particles, the ability to move in a certain period of time decreases and the stem has unstable behavior in the state of PD = 3. By increasing the PD to 5, a slight improvement in displacement has been observed and in PD = 7, it has shown better performance. It can be concluded that with the increasing of density of particle, more momentum of particles will transfer to the polymer membrane. In Fig. 11B displacement of the polymer chain is presented without showing the fluid particles which has achieved more displacement in PD = 7 at t = 8.5 s and compared to the Fig. 3 (PD = 10), PD = 7 has been observed almost 21% less displacement. Also, the study of the parameters of gyration radius and VACF show that less perturbation in chain motion is observed with increasing density or higher PD parameter.

As can easily be resulted in Table 3, by increasing the number of beads, the ratio of velocity and strain (performance) has increased but stronger repulsive parameter has negative effect on performance from velocity and stain ratio aspect. Also, stronger bonding coefficient provides lower ratios (stiff materials) while higher density not only gives higher displacement but also displacement of stem is more stable.

**Table 3:** Ratio of average velocity and strain of polymer chain for different polymer beads number, repulsive, harmonic bonding and density parameter.

Parameter $\frac{Vi}{Vj}$ i, j = 1–3 ($L$ = 20–50)	Parameter $\frac{{\epsilon }_{i}}{{\epsilon }_{{\varvec{j}}}}$ i, j = 1–3 ($L$ = 20–50)
*V4/V1* = *12.50*	${{\epsilon _{4} } \mathord{\left/ {\vphantom {{\epsilon _{4} } {\epsilon _{1} }}} \right. \kern-\nulldelimiterspace} {\epsilon _{1} }}$=1.87
*V4/V2* = *3.98*	${{\epsilon _{4} } \mathord{\left/ {\vphantom {{\epsilon _{4} } {\epsilon _{2} }}} \right. \kern-\nulldelimiterspace} {\epsilon _{2} }}$ = 1.70
*V4/V3* = *1.45*	${{\epsilon _{4} } \mathord{\left/ {\vphantom {{\epsilon _{4} } {\epsilon _{3} }}} \right. \kern-\nulldelimiterspace} {\epsilon _{3} }}$ = 1.38

Parameter $\frac{Vi}{Vj}$ i, j = 1–3 (a = 1, 2, 4)	Parameter $\frac{{\sigma }_{i}}{{\sigma }_{j}}$ i, j = 1–3 (a = 1, 2, 4)
*V3/V1* = *0.54*	${{\epsilon _{3} } \mathord{\left/ {\vphantom {{\epsilon _{3} } {\epsilon _{1} }}} \right. \kern-\nulldelimiterspace} {\epsilon _{1} }}$=0.72
*V3/V2* = *0.81*	${{\epsilon _{3} } \mathord{\left/ {\vphantom {{\epsilon _{3} } {\epsilon _{2} }}} \right. \kern-\nulldelimiterspace} {\epsilon _{2} }}$ = 0.78

Parameter $\frac{Vi}{Vj}$ i, j = 1–5 (S = 4000—8000)	Parameter $\frac{{\epsilon }_{i}}{{\epsilon }_{j}}$ i, j = 1–5 (S = 4000—8000)
*V1/V5* = *1.98*	${{\epsilon _{1} } \mathord{\left/ {\vphantom {{\epsilon _{1} } {\epsilon _{5} }}} \right. \kern-\nulldelimiterspace} {\epsilon _{5} }}$=1.54
*V1/V4* = *1.67*	${{\epsilon _{1} } \mathord{\left/ {\vphantom {{\epsilon _{1} } {\epsilon _{4} }}} \right. \kern-\nulldelimiterspace} {\epsilon _{4} }}$ = 1.41
*V1/V3* = *1.32*	${{\epsilon _{1} } \mathord{\left/ {\vphantom {{\epsilon _{1} } {\epsilon _{3} }}} \right. \kern-\nulldelimiterspace} {\epsilon _{3} }}$ = 1.21
*V1/V2* = *1.18*	${{\epsilon _{1} } \mathord{\left/ {\vphantom {{\epsilon _{1} } {\epsilon _{2} }}} \right. \kern-\nulldelimiterspace} {\epsilon _{2} }}$ = 1.11

Parameter $\frac{Vi}{Vj}$ i, j = 1–3 (PD = 3, 5, 7)	Parameter $\frac{{\epsilon }_{i}}{{\epsilon }_{j}}$ i, j = 1–3 (PD = 3, 5, 7)
*V3/V1* = *3.65*	${{\epsilon _{3} } \mathord{\left/ {\vphantom {{\epsilon _{3} } {\epsilon _{1} }}} \right. \kern-\nulldelimiterspace} {\epsilon _{1} }}$=1.55
*V3/V2* = *1.68*	${{\epsilon _{3} } \mathord{\left/ {\vphantom {{\epsilon _{3} } {\epsilon _{2} }}} \right. \kern-\nulldelimiterspace} {\epsilon _{2} }}$ = 1.33

## Conclusion

In this study, a model for a moving artificial myosin organ was proposed as the smallest moving organ in an artificial muscle. According to this model, the electro-osmotic flow causes the pumping of fluid and by transmitting force to the membrane of the impermeable polymer membrane; it causes swelling and consequently the angular movement of the myosin stem. Initially, the results were validated in a simple channel without the presence of polymer and the DPD method had less than 10% differences with the analytical results. The results were then extended to the proposed model. This mechanism of movement of artificial myosin, which is very close to the natural model, was studied from different aspects including:

- Impact of EOF parameters
- kH parameter: less than 8% raising in polymer displacement by enhancing the ionic concentration (after saturation, there is no displacement was observed)
- Zeta potential: almost 40% increasing in membrane displacement by changing zeta potential from − 25 to − 250 mV (conspicuous displacement was reported)
- Electric field: Increasing the displacement of about 23% due to the increase of the electric field from 50 to 250 V/m (noticeable parameter from controlling parameter during action)
- Impact of DPD fluid and polymer parameters
- Beads number: remarkable reduction of polymer flexibility (20%) with reduction of beads from 50 to 30 (determination of flexibility of membrane)
- Repulsive parameters: decreasing of displacement (16%) by enhancing this parameter (determination of types of DPD fluid)
- Harmony hardness coefficient: significant reduction of polymer flexibility (36%) using 2 times enhancing the harmony hardness coefficient (determination of flexibility of membrane)
- Particle density: Improper performance of artificial myosin with a decrease in particle density from 10 to lower values.

All results including fluid particle displacement, polymer, gyration radius and velocity correlation function were evaluated and compared. It can be concluded that the use of electro-osmotic flow can be used to move myosin and consequently contraction of an artificial muscle.

## Data Availability

The data that support the findings of this study are available from the corresponding author upon reasonable request.
